# Development of a Machine Learning Approach for Local-Scale Ozone Forecasting: Application to Kennewick, WA

**DOI:** 10.3389/fdata.2022.781309

**Published:** 2022-02-10

**Authors:** Kai Fan, Ranil Dhammapala, Kyle Harrington, Ryan Lamastro, Brian Lamb, Yunha Lee

**Affiliations:** ^1^Center for Advanced Systems Understanding, Görlitz, Germany; ^2^Helmholtz-Zentrum Dresden Rossendorf, Dresden, Germany; ^3^Laboratory for Atmospheric Research, Department of Civil and Environmental Engineering, Washington State University, Pullman, WA, United States; ^4^Washington State Department of Ecology, Olympia, WA, United States; ^5^Max Delbrück Center for Molecular Medicine, Berlin, Germany; ^6^Environmental Geochemical Science, School of Science and Engineering, State University of New York, New Paltz, NY, United States

**Keywords:** machine learning, air quality forecasts, ozone, random forest, multiple linear regression

## Abstract

Chemical transport models (CTMs) are widely used for air quality forecasts, but these models require large computational resources and often suffer from a systematic bias that leads to missed poor air pollution events. For example, a CTM-based operational forecasting system for air quality over the Pacific Northwest, called AIRPACT, uses over 100 processors for several hours to provide 48-h forecasts daily, but struggles to capture unhealthy O_3_ episodes during the summer and early fall, especially over Kennewick, WA. This research developed machine learning (ML) based O_3_ forecasts for Kennewick, WA to demonstrate an improved forecast capability. We used the 2017–2020 simulated meteorology and O_3_ observation data from Kennewick as training datasets. The meteorology datasets are from the Weather Research and Forecasting (WRF) meteorological model forecasts produced daily by the University of Washington. Our ozone forecasting system consists of two ML models, ML1 and ML2, to improve predictability: ML1 uses the random forest (RF) classifier and multiple linear regression (MLR) models, and ML2 uses a two-phase RF regression model with best-fit weighting factors. To avoid overfitting, we evaluate the ML forecasting system with the 10-time, 10-fold, and walk-forward cross-validation analysis. Compared to AIRPACT, ML1 improved forecast skill for high-O_3_ events and captured 5 out of 10 unhealthy O_3_ events, while AIRPACT and ML2 missed all the unhealthy events. ML2 showed better forecast skill for less elevated-O_3_ events. Based on this result, we set up our ML modeling framework to use ML1 for high-O_3_ events and ML2 for less elevated O_3_ events. Since May 2019, the ML modeling framework has been used to produce daily 72-h O_3_ forecasts and has provided forecasts via the web for clean air agency and public use: http://ozonematters.com/. Compared to the testing period, the operational forecasting period has not had unhealthy O_3_ events. Nevertheless, the ML modeling framework demonstrated a reliable forecasting capability at a selected location with much less computational resources. The ML system uses a single processor for minutes compared to the CTM-based forecasting system using more than 100 processors for hours.

## Introduction

Chemical transport models (CTMs) are widely used to simulate the temporal and spatial variation of air quality (Sportisse, 2007). Chemical transport models include various physical and chemical processes of the atmosphere as well as known sources and sinks. However, due to the lack of understanding of the important physical and chemical processes in the atmosphere (Seinfeld and Pandis, 2016), CTM simulations can suffer from significant uncertainties and errors, even though the accuracy of numerical models seems to improve over time. Most operational air quality forecast systems are based on CTM and thus can experience systematic biases and errors that result in failure to forecast poor air quality events. In addition, there is a high cost for those forecasts due to the demanding computational requirements and the need for well-trained personnel to operate complex models.

The Air Indicator Report for Public Awareness and Community Tracking (AIRPACT) was developed for air quality forecasting for the Pacific Northwest (PNW) of the United States. AIRPACT, operated by Washington State University, uses the Community Multiscale Air Quality Modeling System (CMAQ) model with Weather Research and Forecasting (WRF) meteorological inputs provided by the University of Washington. The AIRPACT domain covers Washington, Idaho and Oregon along with peripheral areas with 4-km horizontal grid cells and 37 vertical levels. AIRPACT uses the Carbon Bond version 5 (CB05) gas chemistry mechanism and AERO6 aerosol module. It provides 48-h forecasts produced daily, which are available via the web^1^ for the public and local air quality agencies.

Within the AIRPACT domain, Kennewick, Washington (WA) is part of the Tri-cities metropolitan area with a total population of about 220,610 [the combined population of Kennewick (84,960), Pasco (77,100) and Richland (58,550) in 2020] (Washington State Office of Financial Management, 2020). The city is located 32 km north of Washington state's southern border with Oregon and is in a hot and dry portion of the state. Recent monitoring and a large field study have shown that O_3_ mixing ratios can be unhealthy on days in the summer and early fall (Jobson and VanderSchelden, 2017). One EPA Air Quality System (AQS) monitoring site measures the O_3_ mixing ratios at Kennewick, which identified several unhealthy air quality events in 2017 and 2018, while the daily forecasts struggle to identify unhealthy days in this area: e.g., excluding the wildfire affected days (more details will be discussed in Section O_3_ Observations at Kennewick, WA), there were 10 days when the air quality was unhealthy for sensitive groups in 2017–2018, but AIPRACT missed all of them.

Machine learning (ML) models have been used to predict air quality in recent years (e.g., Feng et al., 2015; Freeman et al., 2018; Zamani Joharestani et al., 2019). The numerical air quality models require a huge computational power and many input data, such as the meteorological and emission data over the whole domain. Compared to numerical models, ML methods tend to be more computationally efficient, require less input data, and perform better for specific events. The ML models typically incorporate a variety of features, including observed pollutant levels and various meteorological variables as the basis for training and applying ML methods. For example, Feng et al. (2015) used trajectory-based geographic parameters, meteorological forecasts and associated pollutant predictors as input to an artificial neural network, to predict PM2.5 concentrations in Beijing, China. Freeman et al. (2018) used a recurrent neural network with long short-term memory to predict 72-h O_3_ forecasting using hourly air quality and meteorological data. Zamani Joharestani et al. (2019) tested three machine learning approaches [i.e., random forest (RF), extreme gradient boosting, and deep learning] using 23 features to predict the PM2.5 concentrations in Tehran, Iran.

In this study, we developed a ML modeling framework to predict O_3_ mixing ratios that is based on RF and multiple linear regression (MLR). Random forest is one of the most popular machine learning methods and has been used in air quality modeling and forecast studies. The RF method has been demonstrated to provide reliable forecasts for O_3_ and PM2.5 with lower computational costs compared to physical models (Yu et al., 2016; Rybarczyk and Zalakeviciute, 2018; Zhan et al., 2018; Pernak et al., 2019). Random forest consists of an ensemble of decision trees; decision tree learning is a method for approximating discrete-valued functions (Kam, 1995; Mitchell, 1997; Breiman, 2001). The RF model can be used for classification and regression, but it was suggested that it could lead to the under-prediction of the high pollution events (Jiang and Riley, 2015; Pernak et al., 2019). Since this research aims to provide a reliable O_3_ forecast, especially for the high pollution events, a MLR or second phase RF model is also used to improve the model performance for the high O_3_ predictions to address the under-predictions of a simple RF model. Multiple linear regression is a regression method with one dependent variable and several independent variables. Previous studies that used MLR models to predict O_3_ mixing ratios showed performance that matched more complex machine learning models (Chaloulakou et al., 1999; Sousa et al., 2007; Arganis et al., 2012; Moustris et al., 2012). Yuchi et al. (2019) used RF and MLR for indoor air quality forecasts, and RF provided better predictions for the data in the training dataset, while MLR provided better predictions for conditions that were not represented in the training dataset.

The goal of this study is to develop a reliable air quality forecast framework using machine learning approaches and to apply the system for Kennewick, WA with a focus on the predictability of unhealthy days related to O_3_. Section Dataset and Modeling Framework presents the datasets and the ML forecast framework based on the two machine learning approaches. Section Results and Discussion presents the feature selection, evaluation of the model performance using 10-time, 10-fold cross-validation, and the ensemble forecasts at Kennewick. Finally, Section Conclusions provides conclusions.

## Dataset and Modeling Framework

### Training Dataset of Kennewick

The training dataset for our ML models includes the previous day's observed O_3_ mixing ratios from AQS data, time information (hour, weekday, month), and simulated meteorology from daily WRF forecasts from May to September during 2017–2020 at Kennewick, WA. Because heat and sunlight favor O_3_ generation (Weaver et al., 2009), the observations for the training set only include from May to September. Weather Research and Forecasting meteorological output was obtained from the University of Washington (Mass et al., 2003), which is used in AIRPACT as an input to generate emissions and to drive the CMAQ forecast model. We use temperature (T), surface pressure (P), relative humidity (RH), wind speed, wind direction, and planetary boundary layer height (PBLH) in the training dataset. Time information is included in the training dataset due to the significant patterns of O_3_ variations at diurnal, weekly, and monthly temporal scales.

### Machine Learning Modeling Framework

**Figure 1:**
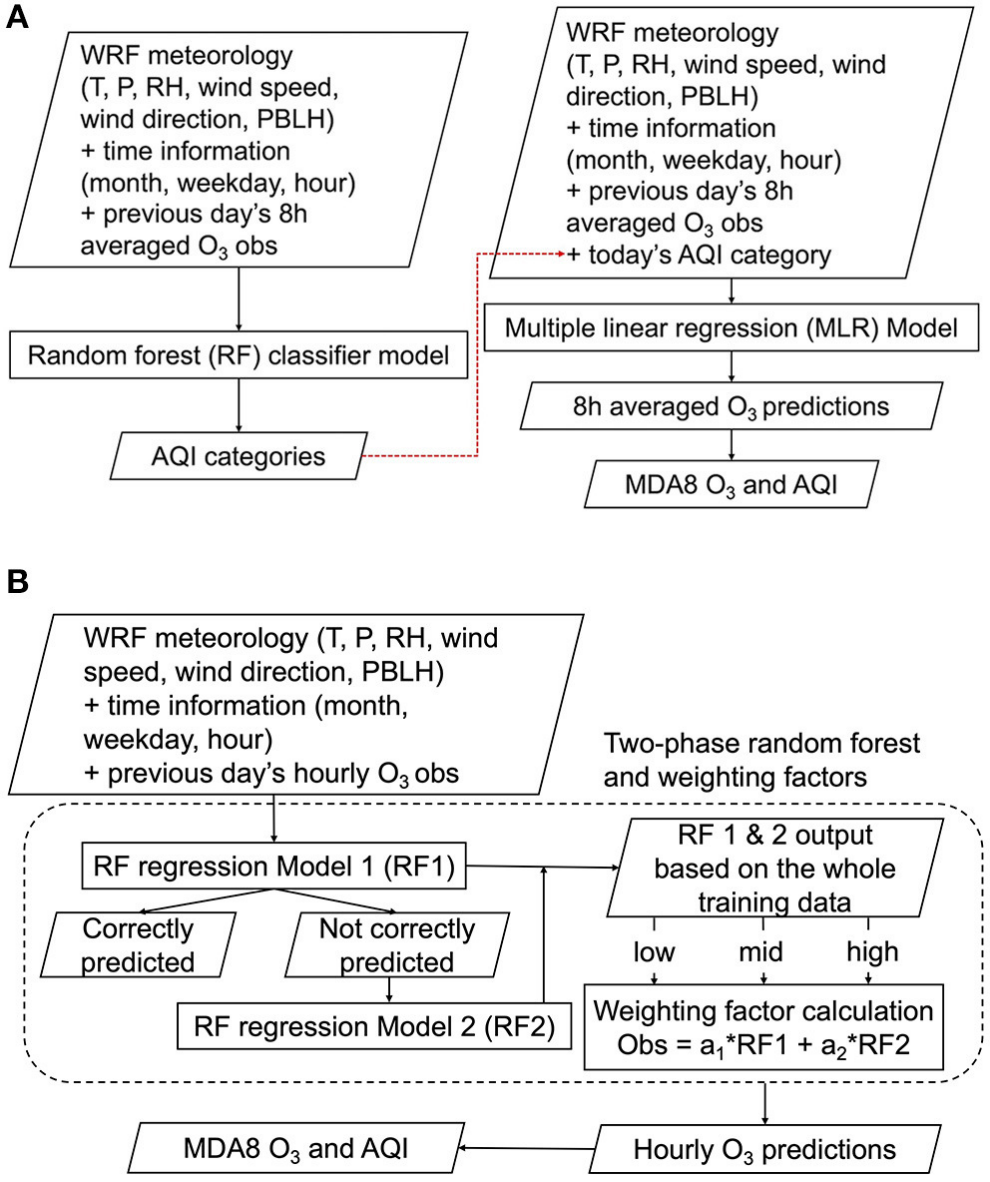
**(A)** ML1 model based on random forest (RF) classifier and multiple linear regression (MLR) models **(B)** ML2 model based on a two-phase RF regression and weighting factors. (MDA8 O_3_: the maximum daily 8-hour moving average O_3_).

We have developed an air quality forecast modeling framework that consists of two independent ML models. The first machine learning model (ML1; Figure 1A) consists of RF classifier and MLR models. The *RandomForestClassifier* and *RFE* functions in the Python module *scikit-learn* were used (Pedregosa et al., 2011). In ML1, the WRF meteorology, time information, and previous day's O_3_ mixing ratios were first used to train a RF classifier model to predict AQI categories. Because the AQI category is based on the 8-h averaged O_3_, the training data for the RF classifier model used the previous day's 8-h averaged O_3_ mixing ratios. Given that a highly polluted episode is generally a rare event, it makes the dataset unbalanced, and the unbalanced training data may produce a bias toward commonly observed O_3_ events (Haixiang et al., 2017). To address this problem, the *balanced_subsample* option was used for the RF classifier. The *balanced_subsample* gives weights to the AQI category values based on their frequency in the bootstrap sample for each tree, so that high AQI values with low frequency in the training dataset are weighted proportionally more: this is an algorithm-level strategy commonly used in machine learning to reduce bias for the majority category for datasets with class imbalance. Without applying this strategy, our machine learning model fitting can be negatively impacted by a disparity in the frequencies of the observed classes (here, AQI classes). Separately, the observed AQI categories were added to the training dataset to train the MLR model (see the red dashed line shown in Figure 1A). When used for forecasting, the RF classifier model was first used to predict the AQI categories, which, in turn, provided input to the MLR model to predict the O_3_ mixing ratios.

The second machine learning model (ML2; Figure 1B) was based on a two-phase RF regression model. Here, the *RandomForestRegressor* function in the Python module *scikit-learn* was used (Pedregosa et al., 2011). ML2 used the WRF meteorology, time information, and previous day's hourly O_3_ mixing ratios to train an RF regression model to predict the concentrations. The entire historical dataset was used to train the first RF regression model (RF1 in Figure 1B). The training data were isolated when RF1 predicted O_3_ mixing ratios that differ from the observations by more than 5 ppb, and then the isolated dataset was used to train the second RF regression model (RF2 in Figure 1B). The training dataset for RF2 was the subset of the entire training data, so RF2 required more decision trees (100 trees for RF1 and 200 trees for RF2). We found that using more decision trees in RF2 led to better performance without significantly increasing the computational cost. Jiang and Riley (2015) also used more decision trees in their second-phase model training (300 trees in the first phase and 500 trees in the second phase). This is why it is called a two-phase RF regression model. In ML2, the final O_3_ mixing ratios are computed using Equation (1) with a set of weighting factors (*a*_1_ and *a*_2_).


(1) $$\begin{array}{r} Hourly\;prediction\;=\;a_{1}\;*\;RF1\;+a_{2}\;*\;RF2 \end{array}$$

The *a*_1_ and *a*_2_ are determined in the training process because the observed ozone data (truth) is available to the models. We divide the *RF*1 ozone predictions into three categories (low, mid, and high) and find the optimal weighting factors at each category using a linear regression equation in Equation (1). When forecasting, *RF*1 and *RF*2 are computed first and then the *RF*1 prediction determines which weighting factors to use and the hourly O_3_ prediction is computed using Equation (1).

### Computational Requirements

Our ML modeling framework requires much less computational power than the AIRPACT CMAQ system. The ML models use a single Intel E5 processor to train and evaluate the model. For the walk-forward cross-validation (more details will be discussed in the Supplementary Materials), ML1 takes about 8 min of CPU time to train the model and to predict daily O_3_ at one location for the entire 2018–2020 ozone season (425 days in total), while ML2 takes about 27 min of CPU time to predict the same time period. These times are much less than AIRPACT that requires 360 h of CPU time (120 Intel E5 processors for 3 h) for a single 48-h forecast.

### Forecast Verifications for AQI Evaluation

Forecast verifications are used to evaluate the machine learning models: Heidke Skill Score (HSS), Hanssen-Kuiper Skill Score (KSS), and Critical Success Index (CSI). Table 1 is a 2 × 2 contingency table that shows a simple unhealthy or good case: “unhealthy” refers to unhealthy air pollution events, and “good” refers to good air quality. Equations (2)–(4) show how HSS, KSS, and CSI are computed (Jolliffe and Stephenson, 2012), where a, b, c, and d refer to the numbers of hits, false alarms, misses and correct negatives, respectively; n refers to the total number of events.


(2) $$\begin{array}{r} HSS=\frac{a+d-a_{r}-d_{r}}{n-a_{r}-d_{r}} \end{array}$$

where $a_{r}=\frac{\left(a+b\right)\left(a+c\right)}{n}$; $d_{r}=\frac{\left(b+d\right)\left(c+d\right)}{n}$


(3) $$\begin{array}{r} KSS=\frac{ad-bc}{\left(b+d\right)\left(a+c\right)} \end{array}$$

(4) $$\begin{array}{r} CSI=\frac{a}{a+b+c} \end{array}$$

Heidke Skill Score represents the accuracy of the model prediction compared with a reference forecast [*r* in Equation (2)], which is from the random guess that is statistically independent of the observations (Wilks, 2011; Jolliffe and Stephenson, 2012). The range of the HSS is from –∞ to 1. A negative value of HSS indicates a random guess is better, 0 indicates no skill, and 1 indicates a perfect score. Hanssen-Kuiper Skill Score measures the ability to separate different categories (Wilks, 2011; Jolliffe and Stephenson, 2012). The range is from −1 to 1 where 0 indicates no skill, and 1 indicates a perfect score. Critical Success Index is the number of hits divided by the total number of forecast and/or observed events (Wilks, 2011; Jolliffe and Stephenson, 2012), which shows the model performance for each category. The range of CSI is from 0 to 1.

**Table 1:** A 2 × 2 contingency table for forecast skill.

**Forecasts**	**Observations**		
	**Unhealthy**	**Good**	**Total**
Unhealthy	a = hits	b = false alarms	a + b
Good	c = misses	d = correct negatives	c + d
Total	a + c	b + d	a + b + c + d

The worst O_3_ level at Kennewick was unhealthy for sensitive groups (AQI 3) during our study period (2017–2020), excluding the days when the air quality was affected by wildfire smoke. For a multi-category case such as in this study [AQI 1—Good, 2—Moderate, 3—Unhealthy for Sensitive Groups], we use the 3 × 3 contingency table in Table 2 (Doswell and Keller, 1990). The skill scores are computed as follows (Jolliffe and Stephenson, 2012).


(5) $$\begin{array}{r} HSS=\left(\sum_{i=1}^{3}p_{ii}-\sum_{i=1}^{3}p_{i}{\hat{p}}_{i}\right)/\left(\left(1-\sum_{i=1}^{3}p_{i}{\hat{p}}_{i}\right)\right) \end{array}$$

(6) $$\begin{array}{r} KSS=\left(\sum_{i=1}^{3}p_{ii}-\sum_{i=1}^{3}p_{i}{\hat{p}}_{i}\right)/\left(\left(1-\sum_{i=1}^{3}p_{i}p_{i}\right)\right) \end{array}$$

(7) $$\begin{array}{r} CSI_{i}=\;n_{ii}\;/\;\left({\sum_{i=1}^{3}n_{i}+\sum_{i=1}^{3}{\hat{n}}_{i}-n}_{ii}\right) \end{array}$$

The *p*_*ii*_ is the sampling frequency when the observed and model predicted AQI is *i*, and *p*_*i*_ and ${\hat{p}}_{i}$ are the observed and model predicted sample frequency when AQI = *i*. The *n*_*ii*_ is the number of hits for AQI_*i*_, and *n*_*i*_ and ${\hat{n}}_{i}$ are the observed and model predicted event numbers when AQI = *i*.

**Table 2:** A 3 × 3 contingency table for forecast skill.

**Model AQI**	**Observed AQI**		
	**1**	**2**	**3**
1	n_11_	n_12_	n_13_
2	n_21_	n_22_	n_23_
3	n_31_	n_32_	n_33_

## Results and Discussion

### O_3_ Observations at Kennewick, WA

**Figure 2:**
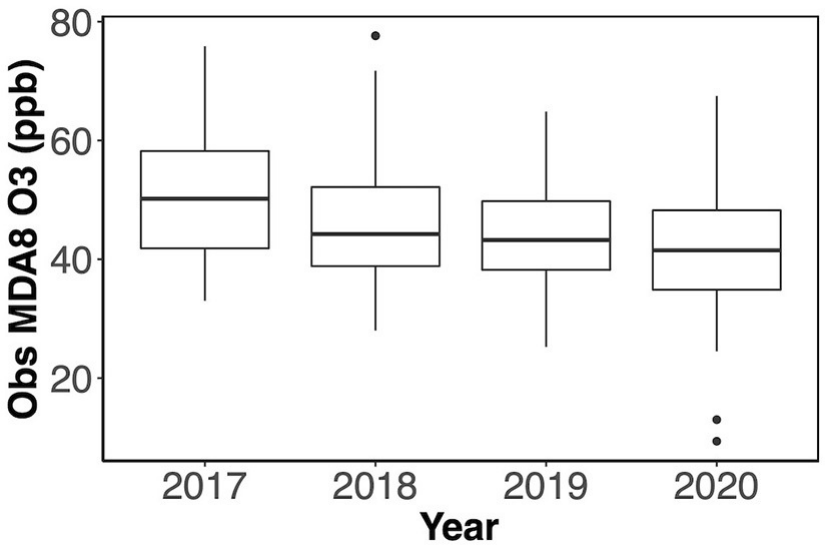
Boxplot of observed (obs) MDA8 O_3_ from May to September in 2017 – 2020.

This research covers the O_3_ observations during the ozone seasons (May–September) from 2017 to 2020. The boxplot in Figure 2 shows that the maximum daily 8-h moving average O_3_ (MDA8 O_3_) observations have decreased from 2017 through 2020. The 2017 and 2018 were fire years, which means they had several regional wildfire events, and there were fewer in 2019 and 2020. The COVID-19 pandemic in 2020 also reduced traffic and other air pollutant emissions.

The Washington State Department of Ecology explored the general relationship between O_3_ level and temperature in the PNW and found that some MDA8 O_3_ was beyond the normal level when the wildfire smoke was presented and there were 4 days identified in 2017–2018: no day identified in 2019–2020. The days affected by wildfire smoke in 2017–2020 have only about 0.75% occurrence rate, which is considered too rare to be predicted well by our ML models. Also, the wildfire smoke effect is not easily predictable, so we exclude these 4 days affected by wildfire smoke from the dataset in this research to avoid the noise brought by the wildfire effects.

Table 3 presents the general statistics of the MDA8 O_3_ observations during the simulated period from May to September in 2017–2020. Here, we define a high-O_3_ day as a day when the observed AQI category is worse than Moderate (i.e., AQI category > 2), which is considered an unhealthy O_3_ event. There are six “high-O_3_ days” for sensitive groups (i.e., AQI category 3) in 2017 and four in 2018. AIRPACT struggled to predict these high-O_3_ days, and it missed all of the 10 “high-O_3_ days”. It is important to note that there were no unhealthy O_3_ events in 2019 and 2020, and the forecasting performance of AIRPACT was better in 2019–2020 than in 2017–2018. It should also be noted that 2020 included potential emission reductions associated with COVID-19 reduced human activities. These emission changes were not incorporated into the AIRPACT emission system. However, ML models implicitly capture changes in emissions when relationships between meteorology and observed O_3_ concentrations are updated during regular re-training (see section Ensemble Forecasts in 2019 and 2020).

**Table 3:** Summary of historical air quality information from May to September in 2017–2020.

**Year**	**Simulated days**	**Mean**	**Median**	**25^**th**^ percentile**	**75^**th**^ percentile**	**# of days for each AQI**			**AQI > 2**
						**1**	**2**	**3**	
2017	100	51	50	42	58	65	29	6	6.0%
2018	148	46	44	39	52	119	25	4	2.7%
2019	136	44	43	38	50	121	15	0	0
2020	142	42	42	35	48	132	10	0	0
Total	526	45	44	38	51	437	79	10	1.9%

*The AQI categories are based on O_3_ mixing ratios only*.

### Machine Learning Model Evaluation at Kennewick WA

Cross-validation is commonly used for machine learning model evaluation by testing on subsets of the data (Raschka, 2015). Among various cross-validation methods available, we use both the 10-time, 10-fold, and walk-forward cross-validation techniques to evaluate our modeling framework. The result from the walk-forward cross-validation methods agrees with the 10-time 10-fold cross-validation, so we present the walk-forward cross-validation results in the Supplementary Materials.

For evaluation purposes, these forecasted hourly or 8-h averaged O_3_ are computed into MDA8 O_3_. We compare the evaluation results of this machine learning modeling framework against the AIRAPCT air quality forecasts for Kennewick, WA. This allows us to test how well this new machine learning-based forecasting system performs with respect to the existing CTM-based modeling framework.

### Feature Selection for Machine Learning Models

We initially provide 10 types of input data for the RF classifier and regression models and 11 types of input data for the MLR model; the additional data in the MLR model is the AQI classification. Since using too many features can cause an overfitting problem (Murphy, 2012), we used the following functions to do feature selection: *feature_importances_* in function *RandomForestClassifier/RandomForestRegressor* and *ranking_* in *RFE*. The selected features were preprocessed by *MaxAbsScaler* in the Python module *scikit-learn* and then used as input to train the model (Pedregosa et al., 2011).

**Figure 3:**
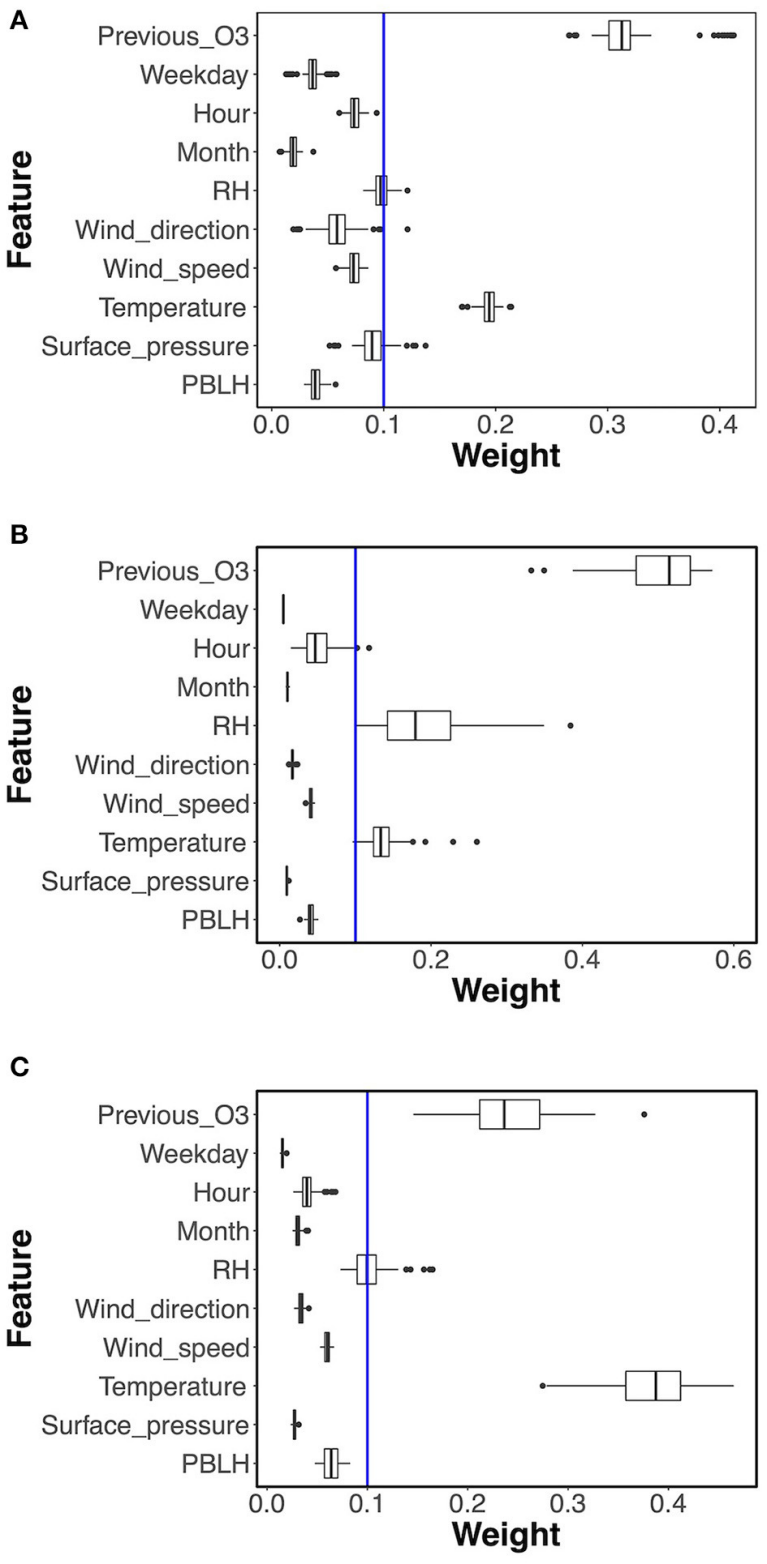
Boxplot of feature weights from **(A)** RF classifier model in ML1, **(B)** the first and **(C)** the second RF regression model in ML2. The blue lines show the mean of the feature weights (0.1).

For the RF classifier model used in ML1 and RF regression model in ML2, the feature selection function with the default setting computed the importance weights, and then the features with weights greater than the mean weight were selected. In this study, the mean weight is 0.1, so only features with weights >0.1 were selected: see the blue lines in Figures 3A–C. Figure 3A shows the weights of the features for the RF classifier model. The feature weights changed in each training process, but the ranking showed very little change. For instance, the previous day's O_3_ observation and temperature were always the selected features, and the relative humidity, surface pressure and wind direction were selected in some cases.

The feature selection results of two-phase RF regression are shown in Figures 3B,C. Similar to the RF classifier model, the previous day's O_3_ observation, temperature, and relative humidity were mostly above the 0.1 weight and thus were selected, but the ranking of the importance weights varied in the two phases. For the first phase RF regression model shown in Figure 3B, the previous day's O_3_ observation was the most important feature, while the relative humidity was more important than temperature. The temperature became the most important feature in the second phase, while the previous day's O_3_ observation ranked second and the relative humidity was selected in some cases.

For the MLR model used in ML1, the built-in feature selection function chose five features, which were AQI category, previous day's O_3_ observation, relative humidity, and surface pressure for all training processes, while the fifth selected feature was either temperature, PBLH, or month.

### 10-Time, 10-Fold Cross-Validation

**Figure 4:**
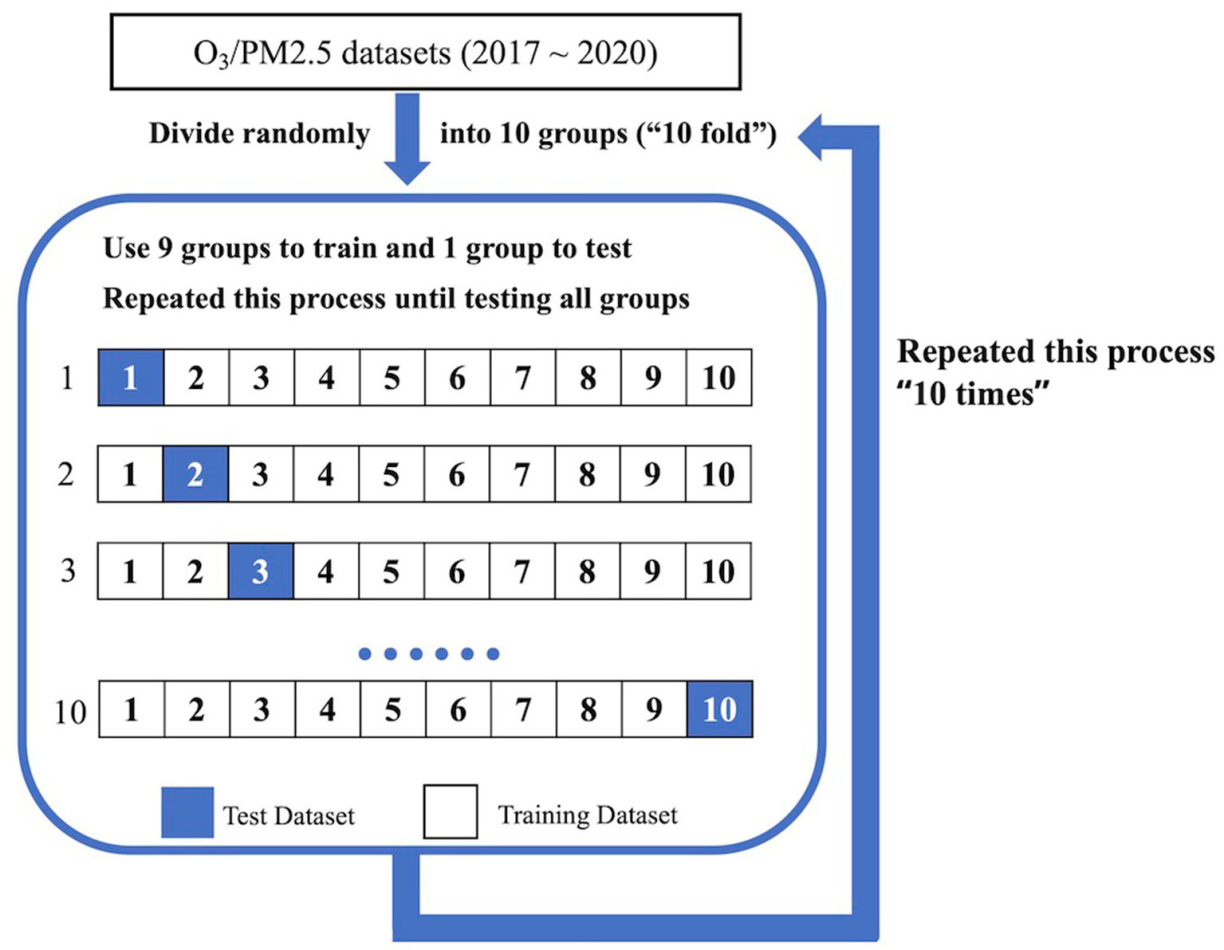
Diagram of 10-time, 10-fold cross-validation.

The *k*-fold cross-validation is one of the most commonly used techniques for machine learning model evaluation (Raschka, 2015). It first divides the dataset into *k* randomly chosen subsets. Then *k* – 1 subsets are used to train the model, while the remaining portion, which is not used in the training process, is used to test the model. This process is repeated k times to test all *k* subsets: every time, the “test” dataset is not used during the training process. In this study, we use *k* = 10, which is termed a 10-fold cross-validation. The *RepeatedKFold* function in the Python module *scikit-learn* is used to separate the dataset (Pedregosa et al., 2011). To avoid any bias from data separation, the 10-fold cross-validation is repeated 10 times (Figure 4) in this research.

The overall performance statistics of the 10-time, 10-fold cross-validations of the O_3_ prediction are presented in Table 4. The mean normalized mean bias (NMB) and normalized mean error (NME) are 5.5 ± 0.2 and 16 ± 0.1% for ML1, −0.14 ± 0.05 and 12 ± 0.1% for ML2, respectively. The low standard deviations show that there is no significant difference between each of the 10 times training conducted, indicating that the model performance is stable. The AIRPACT NMB and NME are 1.1% and 17% when using all data points, which is comparable to the ML performance. Interestingly, AIRPACT has eight extremely over-predicted O_3_ days during the period used in this study. When these extreme values are excluded, its NMB and NME are changed to −2.2% and 14%, respectively. AIRPACT with all data points has a poor correlation (*R*^2^ = 0.070), but without the eight extreme values, the *R*^2^ is 0.38, which becomes comparable to results from the ML models (i.e., *R*^2^ of 0.43 and 0.54). When comparing all models, ML2 has the highest *R*^2^ and the lowest NMB and NME among the three models. We observe similar performance for the ML models using walk-forward method as the 10-time, 10-fold cross-validation (see Supplementary Table 1).

**Table 4:** Statistics and forecast verifications of the 10-time, 10-fold cross-validations of the simulated O_3_ at Kennewick, WA during 2017–2020.

		**AIRPACT**	**AIRPACT (w/o eight**	**ML1**	**ML2**
			**extreme values)**		
*R* ^2^		0.070	0.38	0.43	0.54
NMB (%)		1.1	−2.2	5.2	−0.22
NME (%)		17	14	16	12
HSS		0.34	0.34	0.42	0.4
KSS		0.30	0.30	0.61	0.33
CSI	1	0.85	0.85	0.74	0.87
	2	0.24	0.24	0.34	0.27
	3	0	0	0.28	0

The CSI scores show the model performance for each AQI category. Based on the CSI values, ML2 performs better for the days with AQI 1 (which is the category that most of our O_3_ data fall into), and ML1 performs better for higher O_3_ (AQI > 1). AIRPACT and ML2 do not capture the days with AQI > 2, while ML1 captures 5 out of 10 high O_3_ cases. The better performance of ML1's high O_3_ predictions leads to higher HSS and KSS scores, especially for KSS, which is about two times of AIRPACT and ML2. This makes sense because KSS is sensitive to high-O_3_ events.

ML1 performs better in the high-O_3_ cases, and it is likely due to the linear relationship used in the MLR model that is not as sensitive to the range of data in the training data. Conversely, the RF model in ML2 may not work well when the input data exceeds the range of the training data as it uses an ensemble of decision trees and thus can be limited by the training dataset. The indoor air quality study by Yuchi et al. (2019) that used RF and MLR models drew a similar conclusion.

**Figure 5:**
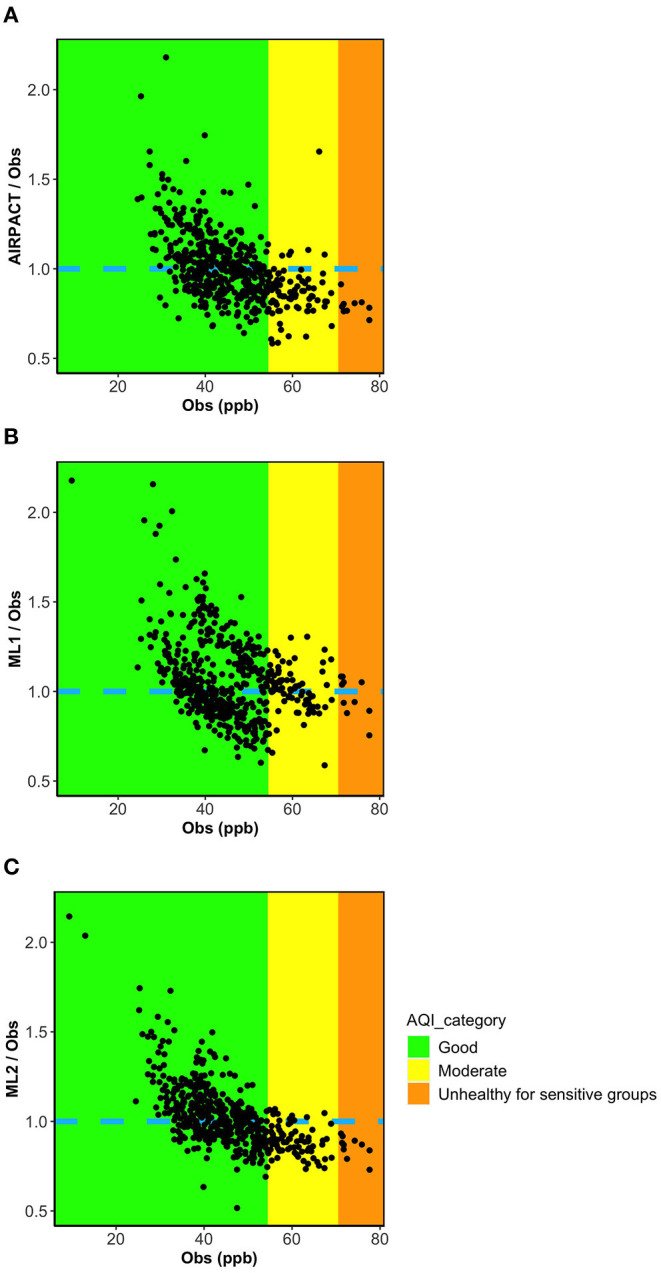
Ratio plots of model prediction to observations vs. observations for three models **(A)** AIRPACT, **(B)** ML1, and **(C)** ML2.

Figures 5A–C show the ratio of the model predictions to the observations vs. the observed MDA8 O_3_ for the AIRPACT, ML1, and ML2 models. To better compare the performance of the three models, the y-axis is set to the same range for all figures, so some extreme values are excluded. Interestingly, all models show a similar systematic bias: over-prediction of low MDA8 O_3_ and under-prediction of high MDA8 O_3_. This figure also shows that ML1 tends to predict higher O_3_ levels than AIRPACT and ML2 for all mixing ratio ranges.

**Figure 6:**
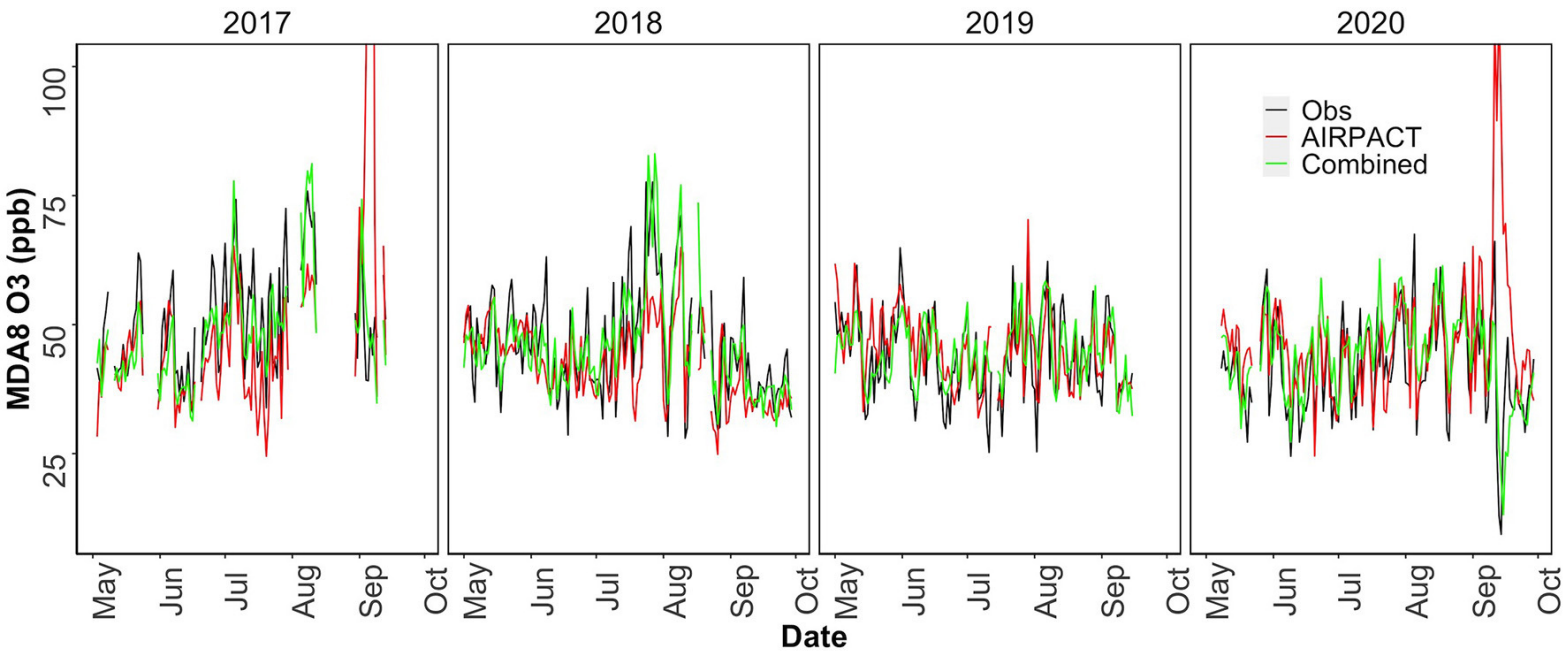
Time series of MDA8 O_3_ from observation, AIRPACT and combined ML model predictions from May to September in 2017–2020.

The results above demonstrate that our ML-based forecasts are comparable to AIRPACT except for the high-O_3_ cases where the ML models clearly perform better. This means the ML models may not outperform the AIRPACT model if there is no high-O_3_ event. Additionally, given the systematic biases shown in Figures 5A–C are strongly associated with the O_3_ levels, the model performance will definitely vary by the distribution of the observed O_3_ levels. Since the average O_3_ levels have decreased from 2017 to 2020 and the year 2019 and 2020 did not have any high-O_3_ event (AQI > 2; see Section O_3_ Observations at Kennewick, WA for the details), we perform the 10-time, 10-fold cross-validations for each year from 2017 to 2020 to explore the changes in the model performances (see Table 5). In addition to the AIRPACT, ML1, and ML2 models, Table 5 includes a “combined” model that is based on our forecast modeling framework that uses ML1 forecasts when the predicted MDA8 O_3_ is higher than 70 ppb and ML2 forecasts for all other cases. The time series of MDA8 O_3_ in Figure 6 shows that both AIRPACT and the combined ML predictions follow the trend of observations. Machine Learning predictions are generally closer to the observations and do not largely over-predict the MDA8 O_3_; however, AIRPACT generates several extremely over-predicted O_3_ events in 2017 and 2020. It should be noted that the “combined” results are available for only 2017 and 2018 because there are no unhealthy O_3_ events in 2019 and 2020, so that only the ML2 model is used for those years.

**Table 5:** Annual statistics and forecast verifications of the 10-time, 10-fold cross-validations at Kennewick, WA.

		**AIRPACT**				**ML1**				**ML2**				Combined^*^	
		**2017**	**2018**	**2019**	**2020**	**2017**	**2018**	**2019**	**2020**	**2017**	**2018**	**2019**	**2020**	**2017**	**2018**
*R* ^2^		0.0053	0.46	0.43	0.029	0.44	0.46	0.34	0.33	0.58	0.64	0.43	0.44	0.57	0.58
NMB (%)		−1.7	−7.5	2.5	12	2.3	4.3	6.6	7.1	−6.3	−1.8	1.4	5.3	−4.5	−0.36
NME (%)		25	14	12	19	15	15	16	18	12	10	11	14	13	11
HSS		0.30	0.31	0.47	0.28	0.41	0.55	0.31	0.31	0.30	0.51	0.32	0.35	0.30	0.52
KSS		0.26	0.22	0.43	0.45	0.45	0.73	0.59	0.77	0.25	0.43	0.24	0.35	0.27	0.44
CSI	1	0.72	0.86	0.90	0.86	0.65	0.81	0.72	0.77	0.73	0.89	0.89	0.91	0.73	0.89
	2	0.24	0.17	0.35	0.23	0.37	0.45	0.27	0.24	0.24	0.35	0.22	0.25	0.14	0.32
	3	0	0	–	–	0.30	0.25	–	–	0	0	–	–	0.30	0.25

* *Combined refers to using the ML1 predicted MDA8 O_3_ predictions for high-O_3_ days and the ML2 predictions for all other days*.

Table 5 shows how the model performance can vary year-to-year due to changes in O_3_ distribution. The changes in the model performance can be explained by the systematic biases trend. As the O_3_ levels go down from 2017/2018 to 2019/2020, the model performance moves from under-prediction to over-prediction: models tend to over-predict the lower O_3_ levels. The walk-forward method performs similarly to the 10-time, 10-fold cross-validation (see Supplementary Figure 1). Compared to the ML models, AIRPACT shows larger variations in the yearly performance, which is likely to be influenced by other changes in the AIRPACT simulations (Munson et al., 2021). The NMB of AIRPACT in 2017 is close to 0 (−1.7%). This is because of its extreme over-prediction in some cases. If they are excluded from the statistics, the NMB of AIRPACT is −12% in 2017. The same reason is attributed to the 12% over-prediction in 2020, and it is 7.3% after removing the extreme predictions. So, excluding the extreme predictions, the NMB from AIRPACT generally reveals the over-prediction of lower O_3_ level and under-prediction of higher O_3_ level. Similarly, ML1 and ML2 show higher NMB in 2019/2020 than in 2017/2018.

Despite these differences, the yearly validation results still show similar performance for the ML models: ML1 performs better for AQI > 2 while ML2 performs better for the other cases. There are unhealthy O_3_ cases (AQI 3) in 2017 and 2018, and ML1 captures half of them. This leads to mostly better statistics than AIRPACT and ML2. The KSS score of ML1 is significantly higher than other models, which is because it is sensitive to the high-O_3_ predictions. ML2 has a good performance for low-O_3_ predictions, and the CSI_1_ and CSI_2_ scores are close or better than AIRPACT. Although the R^2^ values of ML2 decrease in 2019 and 2020, the high CSI_1_ scores (~0.9) still show its accurate low-O_3_ predictions.

ML2 performs better for the low-O_3_ predictions and has higher CSI_1_ scores than ML1, while ML1 can capture more high-O_3_ events with good CSI_3_ scores. The combined approach keeps the high CSI_1_ scores as ML2 and captures some unhealthy O_3_ events in 2017 and 2018. The *R*^2^ of the combined model (*R*^2^ = 0.57 and 0.58 in 2017 and 2018) is better than ML1 (*R*^2^ = 0.44 and 0.46), but slightly worse than ML2 (*R*^2^ = 0.58 and 0.64), because ML2 performs better for the low-O_3_ days that are dominant in the observation datasets.

### Ensemble Forecasts in 2019 and 2020

Beginning in May 2019, the ML modeling framework has been used to provide 72-h “ensemble” operational O_3_ forecasts each day for Kennewick, which uses 27 WRF ensemble forecasts from the University of Washington^2^. The ensemble WRF forecasts use multiple initial and boundary conditions, and various physical parameterizations and surface properties (Mass et al., 2003). We predict O_3_ levels with each WRF member to compute a 72-h forecast and then these individual forecasts are combined to yield an ensemble mean forecast with an associated uncertainty range. The forecasts are available to the public^3^, with the ability to sign up for email alerts if “unhealthy for sensitive groups” or worse AQI levels are forecasted. To increase the size of the training dataset and improve the forecast accuracy, we include the new observational data from the previous day and re-train the models daily.

We present the evaluation of the operational ensemble forecasts covering May to September in 2019 and 2020 in Table 6. The meteorology data used in the cross-validation is extracted from the WRF output that provided input data for AIRPACT, and it is named WRFRT. Most of the statistical variables in Table 6 show that the performance of the ensemble mean is close to the single WRFRT forecasts. By using the ensemble WRF forecasts in the ML forecasting system, the variations of the meteorological forecasts are taken into consideration, although the overall difference between the averaged MDA8 O_3_ and the ensemble members is not significant (within 5%).

**Table 6:** Statistics and forecast verifications in 2019–2020.

		**ML1 (mean)**	**ML1 (WRFRT)**	**ML2 (mean)**	**ML2 (WRFRT)**
*R* ^2^		0.33	0.35	0.49	0.48
NMB (%)		6.9	8.0	5.2	5.7
NME (%)		17	18	12	13
HSS		0.31	0.28	0.41	0.47
KSS		0.64	0.66	0.39	0.44
CSI	1	0.75	0.70	0.90	0.91
	2	0.26	0.24	0.30	0.34

*Note that mean is the ensemble means of the MDA8 O_3_ forecasts of the ensemble members, and WRFRT is the single WRF data that drives AIRPACT*.

**Figure 7:**
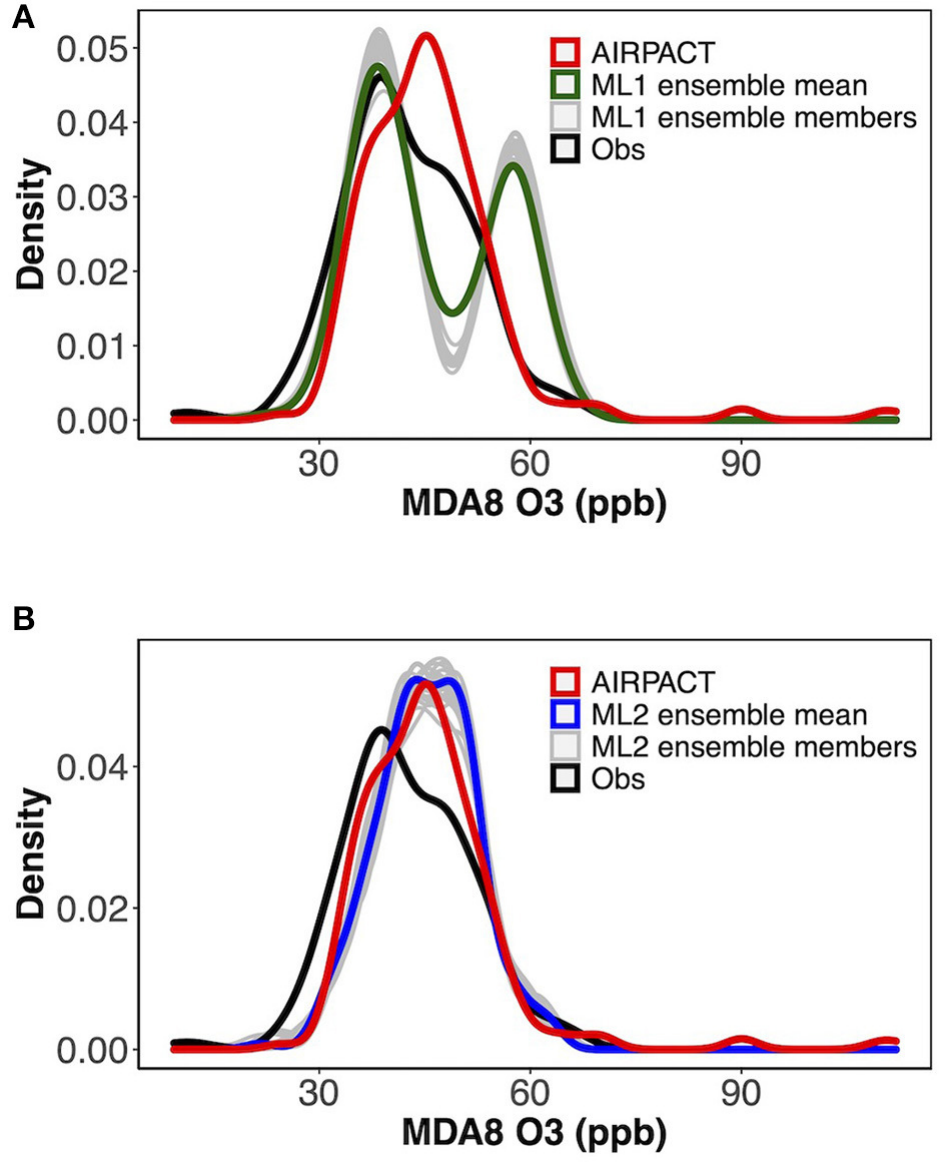
Distributions of observed and **(A)** ML1, **(B)** ML2 model predicted MDA8 O_3_ in 2019 and 2020.

The distributions of the averaged ensemble MDA8 O_3_ predictions are shown in Figures 7A,B. Due to the missing data for some ensemble members, 21 ensemble members are presented in total. The ML1 distributions have two peaks because it first classifies the AQI categories using the RF classifier model. The peaks from ensemble members are higher than the averaged distribution in Figure 7A, and the ensemble-averaged prediction can relatively weaken the bias from a single ensemble WRF member. The distribution of ML2 is close to AIRPACT as shown in Figure 7B. Both ML1 and ML2 do not over-predict MDA8 O_3_ very much, while AIPRACT can severely over-predict some high MDA8 O_3_ events.

## Conclusions

Chemical transport models are widely used for air quality modeling and forecasting, but they may fail to properly forecast pollution episodes, plus they are computationally expensive. AIRPACT is a CTM-based operational forecasting system for the Pacific Northwest, but it has a history of failing to predict high-O_3_ events at Kennewick, WA during summer and fall. In this research, we developed machine learning models that use historical WRF meteorology and O_3_ observation data to build a more reliable forecast system with much less computational burden. The new forecast framework consists of two ML models, ML1 and ML2, that predict the O_3_ mixing ratios and AQI categories. To evaluate and demonstrate this new forecast system, we applied the system to observations from Kennewick, WA over several years.

The O_3_ observations and archived WRF meteorology data (temperature, surface pressure, relative humidity, wind speed, wind direction, and PBLH from 2017 to 2020) were used in the training dataset. ML1 uses both RF classifier and MLR models, and ML2 uses a two-phase RF regression model with weighting factors. The 10-time, 10-fold, and walk-forward cross-validation methods were used to evaluate the modeling framework, and the results agree with each other.

Comparing the statistics of the three models, ML2 has the highest *R*^2^ (0.54) and lowest NMB (−0.22%) and NME (12%). The CSI values from the 10-time, 10-fold cross-validation showed that ML1 performs better for the high MDA8 O_3_ prediction (CSI_3_ = 0.28), and ML2 performs better for the low MDA8 O_3_ predictions (CSI_1_ = 0.87). Given this, our operational forecast system combines ML1 when O_3_ is higher than 70 ppb with ML2 for all other cases.

The ML models provided improved predictions (most *R*^2^ > 0.5) and correctly predicted 5 out of 10 high pollution events, while AIRPACT misses all these events. Also, the model performance of the ML modeling framework was more stable without extreme predictions: AIRPACT predicts eight extremely high MDA8 O_3_ in 2017 and 2020.

Interestingly, we find similar systematic biases from all models; they tend to over-predict the low O_3_ levels and under-predict high O_3_ levels. Due to the systematic biases and decreasing trend of O_3_ from 2017 to 2020, our ML modeling framework performs better than AIRPACT in 2017 and 2018, but shows no improvement in 2019 and 2020. Without unhealthy-O_3_ events in 2019 and 2020, the ML modeling framework cannot demonstrate its superior capability for high O_3_ events.

With about 4 min of CPU time, the ML modeling framework makes it possible to provide the ensemble daily forecast of O_3_ level at Kennewick WA; AIRPACT needs 120 processors for 3 h (360 h of CPU time) throughout the PNW for one single WRF output. The 72-h “ensemble” operational O_3_ forecasts have been provided by this ML modeling framework each day since May 2019. The ensemble mean forecasts take the ensemble model configurations of WRF forecasts into consideration.

Overall, our ML modeling framework is shown to be well-suited for predicting ground-level O_3_ at a specific location using much less computational resources and fewer input datasets than CTMs. Our ML modeling framework has been successfully expanded to predict O_3_ as well as PM2.5 at various AQS sites throughout the PNW region, which will be presented in a subsequent paper. We find that our ML models provide comparable predictability as CTMs (and even excels in some cases) at the locations we have studied (i.e., AQS monitoring sites). However, compared to CTMs, our ML models have a few obvious weaknesses. For instance, ML methods cannot provide predictions over a large domain where there are few monitoring stations, and these methods do not include physical and chemical processes. There are other exciting ML innovations that may help to overcome such weaknesses. We believe ML models can replace CTMs for some specific tasks (e.g., forecasts at specific locations) and a hybrid modeling approach of ML and CTM models could be very beneficial to overcome some of the continuing challenges in traditional atmospheric models.

## Data Availability Statement

The original contributions presented in the study are included in the article/Supplementary Materials, further inquiries can be directed to the corresponding author/s.

## Author Contributions

YL and KH conceptualized the overall study. KF implemented the machine learning models and performed the experiments and validations/analysis with the support of YL, KH, BL, and RD. Datasets used in this study were curated by KF and RD. KF had the lead in writing the manuscript with contributions from YL, and all authors revised the final manuscript. RL participated in this study for a few months as an undergraduate researcher.

## Funding

This work was partially funded by the Center of Advanced Systems Understanding (CASUS) which is financed by Germany's Federal Ministry of Education and Research (BMBF) and by the Saxon Ministry for Science, Culture and Tourism (SMWK) with tax funds on the basis of the budget approved by the Saxon State Parliament.

## Conflict of Interest

The authors declare that the research was conducted in the absence of any commercial or financial relationships that could be construed as a potential conflict of interest.

## Publisher's Note

All claims expressed in this article are solely those of the authors and do not necessarily represent those of their affiliated organizations, or those of the publisher, the editors and the reviewers. Any product that may be evaluated in this article, or claim that may be made by its manufacturer, is not guaranteed or endorsed by the publisher.

## Footnotes

^1^ http://lar.wsu.edu/airpact/

^2^ https://a.atmos.washington.edu/wrfrt/ensembles/info.html

^3^ http://ozonematters.com
