# Supplementary material for: Development of a Machine Learning Approach for Local-Scale Ozone Forecasting: Application to Kennewick, WA
Source: Front Big Data. 2022 Feb 10;5:781309. doi: 10.3389/fdata.2022.781309 (PMC8883518; doi:10.3389/fdata.2022.781309)
Supplement: Supplementary file 1 [file Data_Sheet_1.DOCX]

Supplementary Materials

# Walk-forward cross-validation

Walk-forward cross-validation is a technique to evaluate time-series data. For this, the models are first trained with the 2017 dataset and then used to predict O_3_ for the first day of the 2018 dataset, which is May 1^st^, 2018. Then the May 1^st^, 2018 data is included in the training dataset, and the models are re-trained and used to predict O_3_ for May 2^nd^, 2018. This process is repeated for each successive day of the 2018 – 2020 ozone seasons (May to September).

When a new day’s MDA8 O_3_ is predicted by the ML models, the NMB is recomputed by including the new prediction. The temporal evolution of NMB in **Supplementary Figure 1** shows that the ML models and AIRPACT experience a large variation initially but then tend to somewhat converge. AIRPACT under-predicts the MDA8 O_3_, and the NMB of AIRPACT is decreasing in 2018. However, the model performance of AIRPACT is improved, and its NMB starts to increase after 2018. There is a jump of AIRPACT NMB in the late 2020, which is because of the large over-prediction of some days in September 2020. Both ML1 and ML2 over-predict the MDA8 O_3_, and the NMB of ML1 is higher than ML2. The variation of the NMB from ML models is smaller than AIRPACT, so their performance is more stable by year.

# Supplementary Figures and Tables

## Supplementary Figures

Supplementary Figure 1. Evolutions of the NMB from the walk-forward cross-validation. The black dashed lines mark each year.

## Supplementary Tables

Supplementary Table 1. Statistics and forecast verifications from the walk-forward cross-validations.

|  | | AIRPACT | AIRPACT (w/o 4 extreme values) | ML1 | ML2 |
| --- | --- | --- | --- | --- | --- |
| R^2^ | | 0.15 | 0.35 | 0.4 | 0.51 |
| NMB | | 1.9 | 0.36 | 7.8 | 4.5 |
| NME | | 15 | 13 | 16 | 12 |
| HSS | | 0.34 | 0.31 | 0.35 | 0.47 |
| KSS | | 0.30 | 0.30 | 0.65 | 0.45 |
| CSI | 1 | 0.87 | 0.85 | 0.71 | 0.89 |
|  | 2 | 0.24 | 0.23 | 0.28 | 0.34 |
|  | 3 | 0 | 0 | 0.22 | 0 |

Although the statistics in **Supplementary Table 1** cover three years (2018 – 2020), it generally agrees with the results of the 10-time, 10-fold cross-validation in **Table 4**, which covers four years (2017 – 2020).

# Data Availability Statement

The AQS observation data are available from US EPA^[[1]](#footnote-1)^. The AIRPACT simulation data are available from Laboratory for Atmospheric Research, Washington State University^[[2]](#footnote-2)^. We acknowledge the WRF database from University of Washington^[[3]](#footnote-3)^.

# Code availability

The code discussed in this paper can be found at https://doi.org/10.5281/zenodo.4745320. The GitHub repository, where future updates will be uploaded can be found at https://github.com/kaifan88/ml_kennewick.git.

1. https://aqs.epa.gov/ [↑](#footnote-ref-1)
2. http://lar.wsu.edu/R_apps/ [↑](#footnote-ref-2)
3. https://a.atmos.washington.edu/mm5rt/ [↑](#footnote-ref-3)
